# The citrus flavonoid “Nobiletin” impedes STZ-induced Alzheimer’s disease in a mouse model through regulating autophagy mastered by SIRT1/FoxO3a mechanism

**DOI:** 10.1007/s10787-023-01292-z

**Published:** 2023-08-19

**Authors:** Shohda A. El-Maraghy, Aya Reda, Reham M. Essam, Mona A. Kortam

**Affiliations:** 1 Department of Biochemistry, Faculty of Pharmacy, Cairo University, Cairo, 11562 Egypt; 2 Expanded Programme of Immunization (EPI), Ministry of Health, Cairo, Egypt; 3 Department of Pharmacology and Toxicology, Faculty of Pharmacy, Cairo University, Cairo, Egypt; 4 Biology Department, School of Pharmacy, Newgiza University, Giza, Egypt

**Keywords:** Alzheimer’s disease, Streptozotocin, Nobiletin, EX-527, SIRT1/FoxO3a, Autophagy

## Abstract

The prominence of autophagy in the modulation of neurodegenerative disorders has sparked interest to investigate its stimulation in Alzheimer's disease (AD). Nobiletin possesses several bioactivities such as anti-inflammation, antioxidation, and neuroprotection. Consequently, the study's aim was to inspect the possible neurotherapeutic impact of Nobiletin in damping AD through autophagy regulation. Mice were randomly assigned into: Group I which received DMSO, Groups II, III, and IV obtained STZ (3 mg/kg) intracerebroventricularly once with Nobiletin (50 mg/kg/day; i.p.) in Group III and Nobiletin with EX-527 (2 mg/kg, i.p.) in Group IV. Interestingly, Nobiletin ameliorated STZ-induced AD through enhancing the motor performance and repressing memory defects. Moreover, Nobiletin de-escalated hippocampal acetylcholinesterase (AChE) activity and enhanced acetylcholine level while halting BACE1 and amyloid-β levels. Meanwhile, Nobiletin stimulated the autophagy process through activating the SIRT1/FoxO3a, LC3B-II, and ATG7 pathway. Additionally, Nobiletin inhibited Akt pathway and controlled the level of NF-κB and TNF-α. Nobiletin amended the oxidative stress through enhancing GSH and cutting down MDA levels. However, EX527, SIRT1 inhibitor, counteracted the neurotherapeutic effects of Nobiletin. Therefore, the present study provides a strong verification for the therapeutic influence of Nobiletin in AD. This outcome may be assigned to autophagy stimulation through SIRT1/FoxO3a, inhibiting AChE activity, reducing neuroinflammation and oxidative stress.

**Graphical abstract:**

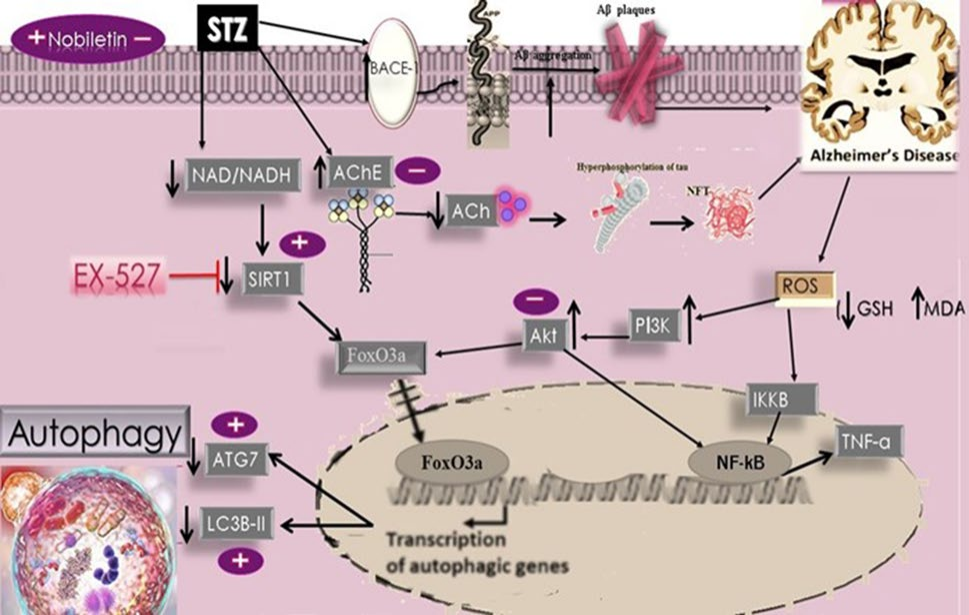

## Introduction

Alzheimer's disease (AD) is an incurable neurodegenerative disorder manifested by behavioral and cognitive deficits which significantly interferes with social and occupational functioning (Tang et al. 2019). As time proceeds, symptoms eventually become severe hindering the patient from doing the daily tasks. Every 5 years after the age of 65, the increased danger of AD doubles (Prince et al. 2013) and may contribute to 60–70% of dementia cases (Garre-Olmo 2018). AD is characterized by three crucial hallmarks which are loss of neuronal cells, the appearance of amyloid-β (Aβ) plaques, and the neurofibrillary tangles (NFTs) existence.

Intracerebroventricular (ICV) injection of streptozotocin (STZ) in rodents has been shown to develop a cognitive behavior deficit, long-term progressive learning, and memory loss that results in oxidative stress, neuroinflammation, and biochemical changes. It is recognized as a reliable experimental animal model which involves the peculiarities that specifies the pathogenesis of neurological disorders. The pathological changes of STZ that cause neuropsychiatric changes, dementia, and eventually neuron death are tau protein and Aβ aggregations (Ravelli et al. 2017).

Autophagy is one of the most rapidly growing factors that showed great importance in neuronal health and development. Accordingly, ample attention is being paid to the molecular mechanisms by which autophagy confines to neurodegenerative diseases. Insufficiency of the crucial autophagy-related gene 5 (ATG5) or ATG7, especially in the central nervous system, resulted in accelerated irregular intracellular protein accumulation, neuronal degeneration, and an incredible amount of tau proteins aggregates, all of which add value to the late onset of many neurodegenerative diseases, which include AD (Hara et al. 2006).

Silent information regulator proteins (SIRT) (sirtuins) are category III histone deacetylases that function as nicotinamide adenine dinucleotide (NAD^+^)-dependent deacetylases. Seven sirtuins (SIRT1–7) are located in various cellular components in mammals. SIRT1 is protein deacetylase ubiquitously present in regions associated with neurodegenerative progressions, including the hippocampus, which supports growth and maintains longevity (Anekonda and Reddy 2006). Furthermore, SIRT1 has the ability to deacetylate both non-histone and histone substrates including Forkhead box-containing protein, O subfamily (FoxO) (Gan 2008). Deacetylation increases the activity of FoxO3a-a mediator of autophagy-resulting in the several target genes expression through intensifying the transcription of several ATGs and regulator genes; for instance, microtubule-correlated proteins 1A/1B light chain 3B (LC-3), Beclin-1, ATG5, ATG7, ATG12, and ATG14 (Zhao et al. 2007; Ferguson et al. 2015). SIRT1 can also establish a molecular complex with a number of key components of autophagy pathways, including the autophagy gene ATG7 (Lee et al. 2008).

Recently SIRT1 revealed to repress beta-secretase (BACE1) activity in several in vitro models, hence relegating the secretion of Aβ (Wang et al. 2013). SIRT1 insufficiency also reduces Akt action by preventing its phosphorylation (Wang et al. 2011).

On the other hand, the inflammatory hierarchy has been a target for SIRT1 as a way of defending Aβ toxicity. This was achieved by impairing nuclear factor-kappa B (NF-κB) and its signaling pathways, including tumor necrosis factor (TNF-α) (Tilstra et al. 2011; Yeung et al. 2004). Moreover, SIRT1 is capable of regulating the level of p-tau via deacetylation and consequently relegates its level (Li et al. 2007). Taken all together, AD has been observed to be closely linked to the deficit of SIRT1 (Ma et al. 2020; Julien et al. 2009) which offered us some insights to mitigate AD through SIRT1 activation.

Nobiletin (5,6,7,8,3′,4′-hexamethoxyflavone) is a flavonoid concentrated in citrus peel that has been shown to have pharmacological effects against cardiovascular and metabolic abnormalities (Eguchi and Murakami 2006). It has a variety of benefits, including antioxidant, anti-carcinogenic (Aoki et al. 2013), anti-inflammatory (Malik et al. 2015; Zhang et al. 2016), and anti-diabetic (Umeno et al. 2016) activities. Nobiletin increases the protein expression of SIRT1/FoxO3a, which regulates autophagy, mitochondrial dynamics, and biogenesis. Abundant studies demonstrated several autophagy-mediated mechanisms of Nobiletin (Jiang et al. 2018; Dusabimana et al. 2019; Wang et al. 2020). However, the exact mechanism by which Nobiletin tends to regulate autophagy through the SIRT-1/FoxO3a pathway in AD remained unrevealed.

Accordingly, this study provided a major opportunity to advance the understanding of the role of Nobiletin to counteract the neurodegeneration that occurs in STZ-induced AD through investigating its underlying molecular mechanisms mastered by autophagy induction (Table 1).

**Table 1:** The measured parameters and their main effects on cognitive behavior impairment, neuroinflammation, and autophagy as a characteristic of STZ-induced AD

The measured parameter	Main effect
BACE1	Responsible for cleavage of the amyloid precursor protein beta site in the brain to generate the amyloid beta peptide
Aβ42	AD has been indicated by cerebral extracellular plaques, created from a compact proteinaceous core enclosing the Aβ peptide
NF-κB	Binding of Aβ aggregates to astrocyte receptors leads to the activation of downstream target genes nuclear factor-κB (NF-κB)
TNF-α	Neuroinflammation can be indicated by estimating NF-κB and their downstream effector, TNF-α
SIRT1	Stimulates autophagy by preventing acetylation of proteins required for autophagy
p-Akt	Akt tends to phosphorylate acetylated FoxO, which ensued a cytoplasmic retention due to binding to 14-3-3 protein that hinders FoxO transcriptional activity
FoxO3a	A mediator of autophagy intensifies the transcription of several ATGs such as ATG5, ATG7, ATG12, and ATG14, as well as LC3B, and Beclin1
LC3B-I, LC3B-II	The ratio of LC3B-II to LC3B-I levels reflects the level of autophagic activity
ATG7	Insufficiency of ATG results in accelerated irregular intracellular protein accumulation, neuronal degeneration, and an immense aggregates, all of which add value to AD
p-tau	Tau protein is a soluble microtubule-associated protein (MAP) which is present in a hyperphosphorylated state in paired helical filaments in AD

*BACE1* beta-secretase 1, *Aβ42* amyloid-β 42 plaques, *NF-κB* nuclear factor-kappa B, *TNF-α* tumor necrosis factor-alpha, *SIRT1* silent information regulator protein 1 (Sirtuin 1), *p-Akt* phosphorylated protein kinase B, *FoxO3a* Forkhead box-containing protein, *O3a, LC3B* microtubule-correlated proteins 1A/1B light chain 3B, *ATG7* autophagy-related gene 7, *p-tau* phosphorylated tau

## Materials and methods

### Animals

Forty male Swiss albino mice weighing 20–25 g were ordered from the animal facility of Faculty of Pharmacy, Cairo University (Cairo, Egypt). Mice were given a 1-week acclimatization prior to the study and were kept in regulated environments with a constant temperature (24 ± 2 °C), light/dark cycle (12/12 h), and relative humidity of 60 ± 10%. During the experiment, mice had unrestricted access to food and water. The research was held with the permission of the Animal Experimentation Research Ethics Committee (Permit Number: BC 2707) in compatible with the National Institutes of Health's Guide for the Care and Use of Laboratory Animals (NIH Publication No. 85-23, revised 2011).

### Drugs

STZ, Nobiletin, and a specific SIRT1 inhibitor (EX-527) were ordered from Sigma-Aldrich Chemical Co. (St. Louis, MO, USA). Nobiletin and EX-527 solutions were prepared by dispersing in 0.5% dimethyl sulfoxide (DMSO) obtained from Sigma-Aldrich Chemical Co. (St. Louis, MO, USA). Unless otherwise specified, all other chemicals were purchased from Sigma-Aldrich (St. Louis, MO, USA). Solutions were prepared daily.

### Experimental design

#### Induction of Alzheimer

AD-like pathological dysfunction was induced in mice using STZ (3 mg/kg) once (Hindam et al. 2020). STZ was freshly prepared in saline solution (0.9% NaCl) and administered ICV by means of the freehand technique (Pelleymounter et al. 2002) renovated by Warnock (2010). Mice displayed normal behavior within 1 min following the administration.

#### Groups

**Scheme 1:**
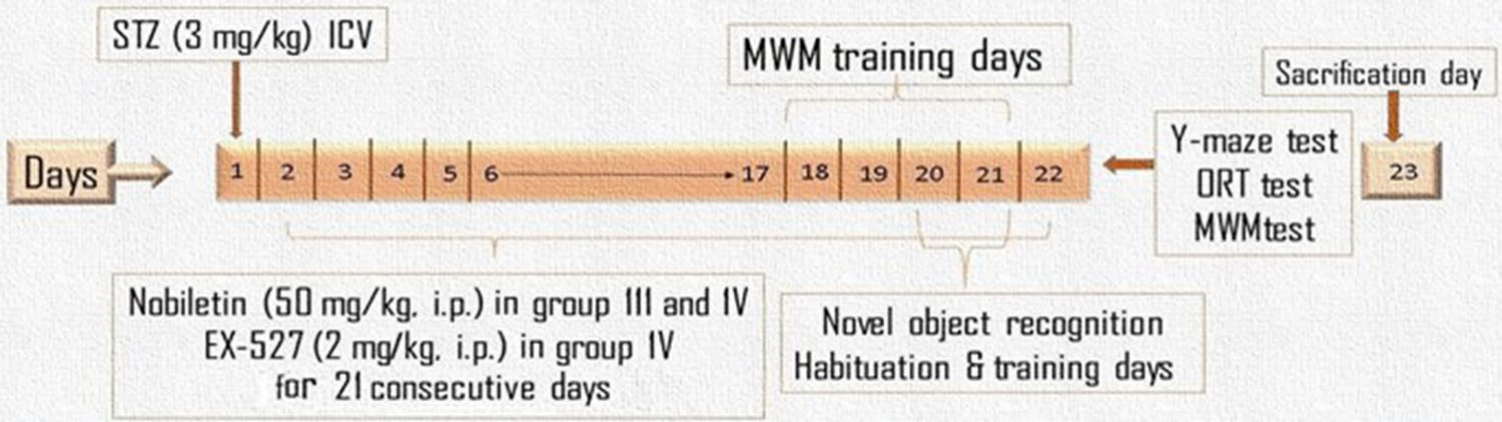
Experimental time line for the administration of Nobiletin, EX-527 and the behavioral assessments in STZ-AD mice model

The mice were categorized into four groups of ten mice each. Group I functioned as the control group, receiving saline and 0.5%DMSO both (ICV) and (i.p.), respectively, and served as the normal group. Group II acted as STZ-AD group where animals injected STZ (3 mg/kg, ICV), once to induce AD-like pathology. Group III was the Nobiletin group where mice received Nobiletin (50 mg/kg, i.p.) (Braidy et al. 2017; Nakajima and Ohizumi 2014) dissolved in 0.5% DMSO, starting 1 day after STZ, and for 21 days. Animals of Group IV were given EX-527 (2 mg/kg, i.p) which was dispersed in 0.5% DMSO 30 min before Nobiletin, for 21 consecutive days (Daenthanasanmak et al. 2019) starting 1 day after STZ administration. Experimental design is demonstrated in Scheme 1.

### Behavioral assessments

Twenty-four hours after the last injection of Nobiletin, spatial learning, memory and cognitive deficits were evaluated using:

#### Novel object recognition test

Object recognition test (ORT) exploited to estimate long-term memory (LTM), learning and assess cognition. The apparatus consisted of a painted wood small chamber with 40 cm × 40 cm × 40 cm dimensions.

The ORT required 3 days to be completed: habituation, training, and testing. During habituation, each mouse was positioned in the center of an exposed, empty arena and given 5 min to freely explore it. Throughout the training day, the mouse was enabled to explore two similar objects for 5 min.

Probe day was 24 h after training where two dissimilar objects were present, a familiar and a novel one. The objects were wiped with 10% ethanol between trials to exclude any odor guidance. Exploration was described by pointing the nose to the object at a distance of about 2 cm and/or touching it. Standing on the object was not considered exploration (Lueptow 2017). The aforementioned variables were tested during a 5-min observation period:

Discrimination index (DI): [time exploring the novel object (s) − time exploring the familiar object (s)]/[time exploring novel (s) + familiar (s)] × 100%.

Recognition index (RI): time exploring novel object/total time of exploration of both objects] × 100%.

#### Y-maze spontaneous alternation test

Y-maze test was conducted to evaluate the short-term memory (STM) and working memory through measuring the spontaneous alternation manners. The device is y-shaped with dimensions 32 cm × 10 cm × 26 cm (Wilcock et al. 2004). Percentage of alternation can be assessed by enabling mice to explore all three arms of the maze (Melnikova et al. 2006). Mice were positioned in the maze's center and enabled to explore all arms for 8 min; during this time, the number and sequence of arm options were measured. The following are the results of spontaneous changes:

The alternation percentage = [(number of alternations)/(number of entries − 2)] × 100, where the number of alternations represents three different successive arms entries such as ABC, BCA, and CAB. After each animal, diluted ethanol was used to remove any olfactory cues (Miedel et al. 2017).

#### 2.4.3. Morris water maze test

The Morris water maze test (MWM) is utilized to examine spatial memory and learning in laboratory animals. MWM comprises a circular pool [90 cm (diameter) × 45 cm (height)], categorized into four quadrants where a colored platform [6 cm (diameter) × 29 cm (height)] was submerged 1 cm underneath the water surface in one of the four quadrants, day 18 was a training to swim for 60 s without the platform. Throughout the following 3 days, the mouse had three trials every day including the platform immersed in the pool. Each mouse was allowed to rest on the platform for 10 s once reaching it. Latency, or the time it took to find the hidden platform, was recorded (Bromley-Brits and Deng 2011). Mice were exposed to a probe session 1 day after the final training session; during this time, the platform was removed, and the time spent by each mouse in the target quadrant was documented as an indicator of memory (Morris 1981).

### Brain processing and tissue sampling

Mice were ultimately euthanized by cervical dislocation under light anesthesia utilizing thiopental sodium (30 mg/kg) after the behavioral tests (day 23) (Ahiskalioglu et al. 2018), and brains were quickly dissected and isolated. Three brains were divided into two hemispheres, the first three hemispheres were preserved in 10% (v/v) neutral buffered formaldehyde for 24 h prior to histopathological staining and immunohistochemistry. The hippocampi of the other three hemispheres were separated and preserved at − 80 °C for Western blot analysis. Hippocampi separated from the remaining brains were preserved at − 80 for the preparation of 10% homogenate to be used for the estimation of ACh, Aβ42, NF-κB, and TNF-α using enzyme-linked immunosorbent assays (ELISA), AChE and BACE-1 enzymes activity, and reduced glutathione (GSH) and malondialdehyde (MDA) levels. Finally, the dead bodies and animal wastes were frozen until they were incinerated.

### Biochemical measurements

#### Estimating hippocampal acetylcholinesterase (AChE) and beta-secretase 1 (BACE1) activity

AChE activity was assayed using the AChE Microplate Assay Kit (cat#: MBS8243242) obtained from My BioSource Inc. (San Diego, CA, USA). The AChE activity was detected by measuring the conversion of acetylthiocholine iodide to thiocholine, which interacts with 5,5-dithiobis-2-nitrobenzoic acid (DTNB) to construct the colorimetric product 5-thio-2-nitrobenzoic acid (TNB), which is commensurate to the AChE activity (Ellman 1959). A 412 nm wavelength was used to detect the color of the product. Units per milligram of protein (U/mg protein) were used to demonstrate the findings.

The total activity of BACE1 present in hippocampus was detected utilizing commercially available secretase-kits from ABNOVA (cat#: KA0900, Taipei, Taiwan) according to the manufacture’s protocol. The fluorometric reaction, conveyed as relative fluorescence U/μg protein, correlates with the level of secretase enzymatic activity.

#### Quantification of hippocampal ACh, Aβ42, NF-κB, and TNF-α levels using ELISA technique

The previously prepared homogenates were used to determine ACh (cat#: MBS265771), Aβ42 (cat#: MBS265825), NF-κB (cat#: MBS043224), and TNF-α (cat#: MBS825075) levels using the corresponding mice ELISA kits obtained from My BioSource Inc. (San Diego, CA, USA). The techniques were accomplished consistent with the manufacturer’s guidelines. The results were represented as pg/mg protein in all parameters. The protein content of tissue homogenate was detected by the means of Bradford assay (Bradford 1976).

#### Western blot analysis for the assessment of SIRT1, pS473-Akt, t-Akt, FoxO3a, LC3B-I, LC3B-II, and ATG7

Tissues were rinsed in phosphate buffered saline (PBS) before being lysed with radioimmunoprecipitation assay (RIPA) lysis buffer supplied by Bio BASIC INC (Markham, Canada). Following that, the lysates were boiled for 5 min in Laemmli buffer. Then proteins were separated by 10% SDS-PAGE (sodium dodecyl sulfate–polyacrylamide gel electrophoresis) and relocated to an immobilon^®^ membrane (Millipore). Protein expressions were assessed utilizing primary antibodies obtained from ThermoFisher Scientific (MA, USA) against SIRT1 (1:1000; cat#: PA5-17074), pS473-Akt (1:250; cat#: 700392), total-Akt (Phospho-Akt1/Akt2/Akt3) (Ser473) (1:500–1:2000; cat#: PA5-99331), FoxO3a (1:1000; cat#: PA5-20973), LC3B-I &LC3B-II (1:1000; cat#: PA1-46286), ATG7 (1:1000; cat#: PA5-17216), and β-actin (1:1000; cat#: MA5-15739). Antibodies were diluted in 5% skimmed milk, Tris–HCl, 0.1% Tween 20, inserted to polyvinylidene difluoride membranes (PVDF), then protein plots were incubated at 4 ℃ overnight. After rinsing, the membrane was incubated with a horseradish peroxidase-labeled secondary antibody for 1 h at room temperature (1: 5000). Protein levels were determined using the Bradford protein assay kit from Thermo Fisher Scientific (MA, USA). According to the manufacturer's instructions, a Bradford test was performed (Bradford 1976). A ChemiDoc imaging system with Image LabTM 6.1 Software was used to examine the band's intensity (Bio-Rad Laboratories Inc., Hercules, California, USA). After being normalized against the β-actin protein, the values were presented as arbitrary units.

#### Estimation of oxidative stress biomarkers

Using a specific Bio-diagnostic kit (cat#: MD2529; Giza, Egypt), oxidative stress status in hippocampal homogenates was measured by evaluating thiobarbituric acid reactive substances (TBARS). The techniques include a reaction of thiobarbituric acid (TBA) and MDA in an acidic medium at 95 °C for 30 min to produce a pink product that can be evaluated at 534 nm, according to Mihara and Uchiyama's method (Mihara 1978). In addition, reduced GSH was assessed according to Ellman’s (1959) protocol, using Bio-diagnostic kit (cat#: GR2511; Giza, Egypt). The method depends on a chromogen yield that is directly proportional to GSH concentration and its absorbance can be measured at 405 nm.

#### Histopathological examination

Specimens were trimmed and treated in serial grades of ethanol, cleared in xylene, infiltrated, and embedded in Ted Pella, Inc. paraplast tissue embedding media (CA, USA). A rotatory microtome was used to cut 4 μm thick sagittal brain sections to demonstrate hippocampal regions in multiple specimens. As a general microscopic analysis staining method, the tissue samples were then inspected with hematoxylin and eosin (H&E) staining under a light microscope (Leica Microsystems GmbH, Wetzlar, Germany).

#### Immunohistochemical detection of p-tau

5 microns’ thick paraffin embedded tissue section was prepared. Deparaffinized retrieved tissue sections were treated with 0.3% H_2_O_2_ for 20 min. Brain samples were incubated with Anti-Phospho Tau (Ser214) (1:100—Thermofisher scientific—cat#: 44-742G) overnight at 4 °C. Tissue sections were washed out by PBS followed by incubation with secondary antibody HRP (horseradish peroxidase) (DAKO Envision™ + System, HRP) obtained from Biocompare (CA, USA) for 20 min, and then it was counterstained with hematoxylin, and cleared in xylene before undergoing microscopic examination. To calculate the area percentage of p-tau immunoexpression levels, six non-overlapping fields were randomly chosen and inspected from each sample's hippocampal dentate gyrus (DG) region. The Leica Implementation module for histological analysis, which was linked to a Full HD microscopic imaging system, was used to gather all light microscopic examinations and data (Leica Microsystems GmbH, Germany).

During the analysis of samples, the researcher was blinded to the sample identity, and sample coding and decoding were accomplished by an independent test. All analysis was done over a region representing 50 μm.

### Statistical analysis

Data were expressed as mean ± standard deviation. A one-way analysis of variance (ANOVA) was used for all parameters, along with Tukey’s multiple comparison test. For statistical analysis, instant automated software (GraphPad Prism software (version 7.4) Inc., San Diego, CA, USA) was employed. For all data analysis, the significance level was established to *p* value < 0.05.

## Results

### Effect of Nobiletin on behavior and memory deficits in STZ-induced AD in mice

Behavior and memory deficits are the foremost features of AD models, which denote hippocampal deterioration and cognitive impairment.

**Fig. 1:**
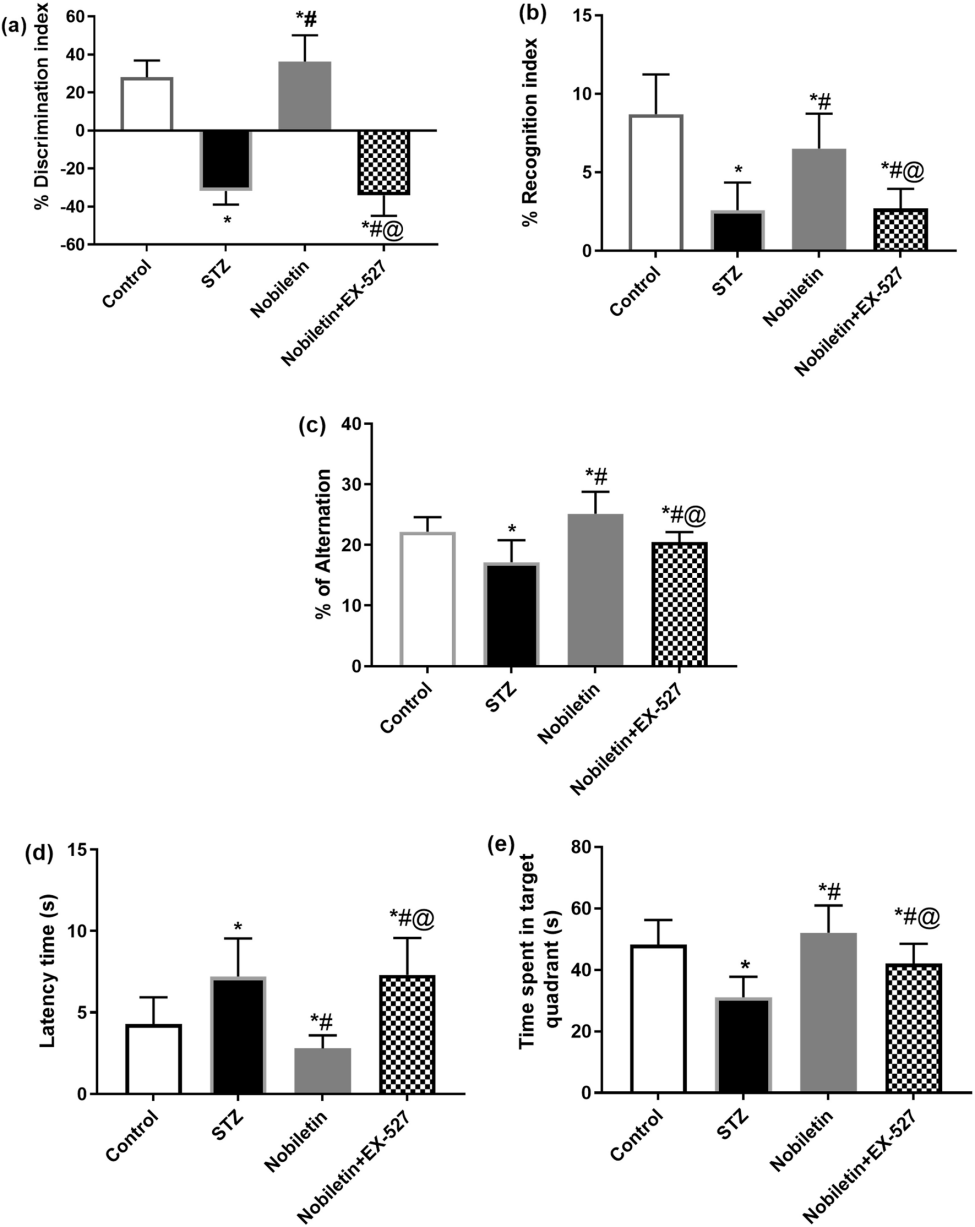
Administration of Nobiletin ameliorated STZ-induced cognitive memory impairment in mice: ORT: **a** the percent discrimination index, **b** the percent recognition index, Y-Maze: **c** the percent of alternation, and MWM: **d** the latency time, (**e**) the time spent in target quadrant. The values are expressed as mean ± S.D. (*n* = 10) for each group. Using one-way ANOVA and Tukey’s post hoc test, values are statistically sig. at *p value < 0.05 vs. the control group, ^#^*p* value < 0.05 vs. the STZ group, and ^@^*p* value < 0.05 vs. the Nobiletin group

In ORT, ICV injection of STZ caused 212.8% suppression in discrimination index and 70.2% reduction in recognition index when compared to control indicating memory and cognitive impairment. In contrast, administration of Nobiletin augmented the discrimination index by approximately 214.7% and recognition index by 2.5-fold when compared to diseased mice. However, EX-527 displayed 193.6% diminution and 0.41-fold decline relative to Nobiletin group in discrimination and recognition indices, respectively, thus blocking Nobiletin effect. These results indicated that Nobiletin-treated mice have a predilection to the novel object over the familiar one (Fig. 1a, b).

In Y-maze test, STZ group indicated significant decrease in percent alternations by 0.77-fold when contrasted to their control counterparts. Mice treated with Nobiletin demonstrated significant change in percent alternation when compared with STZ-AD group by about 47% upsurge. Moreover, EX-527 blocked effect of Nobiletin causing 19% decrease in percent alternation than Nobiletin group (Fig. 1c).

In MWM, the spatial learning progression denoted by latency time showed 1.67-fold increment in STZ-AD mice when contrasted to control group. In the probe test, STZ-AD mice spent less time in the target quadrant (0.64-fold less than control mice). However, Nobiletin proficiently countered these effects, as revealed by 60.9% reduction in latency time as well as boosting the time spent in target quadrant by 1.67-fold when compared to STZ group. Using EX-527 canceled the effect of Nobiletin causing 2.59-fold elevation in latency time and 19.4% reduction in time spent in quadrant when contrasted to Nobiletin group (Fig. 1d, e).

### Effect of Nobiletin on hippocampal AChE activity, ACh, BACE1 activity, and Aβ42 in STZ-induced AD in mice

Mice that received ICV-STZ exhibited a considerable increase in hippocampal AChE activity by 3.28-fold and a 0.47-fold decline in ACh levels as compared to the control group. As for BACE1 activity, STZ triggered BACE1 activity by 3.5-fold and Aβ42 levels by 3.6-fold in comparison to control. On the contrary, the increment in AChE activity was mitigated in Nobiletin-treated mice by 45.8% as contrasted to STZ-AD mice and the effect of STZ was ameliorated by Nobiletin which enhanced ACh levels by approximately 80%.

**Fig. 2:**
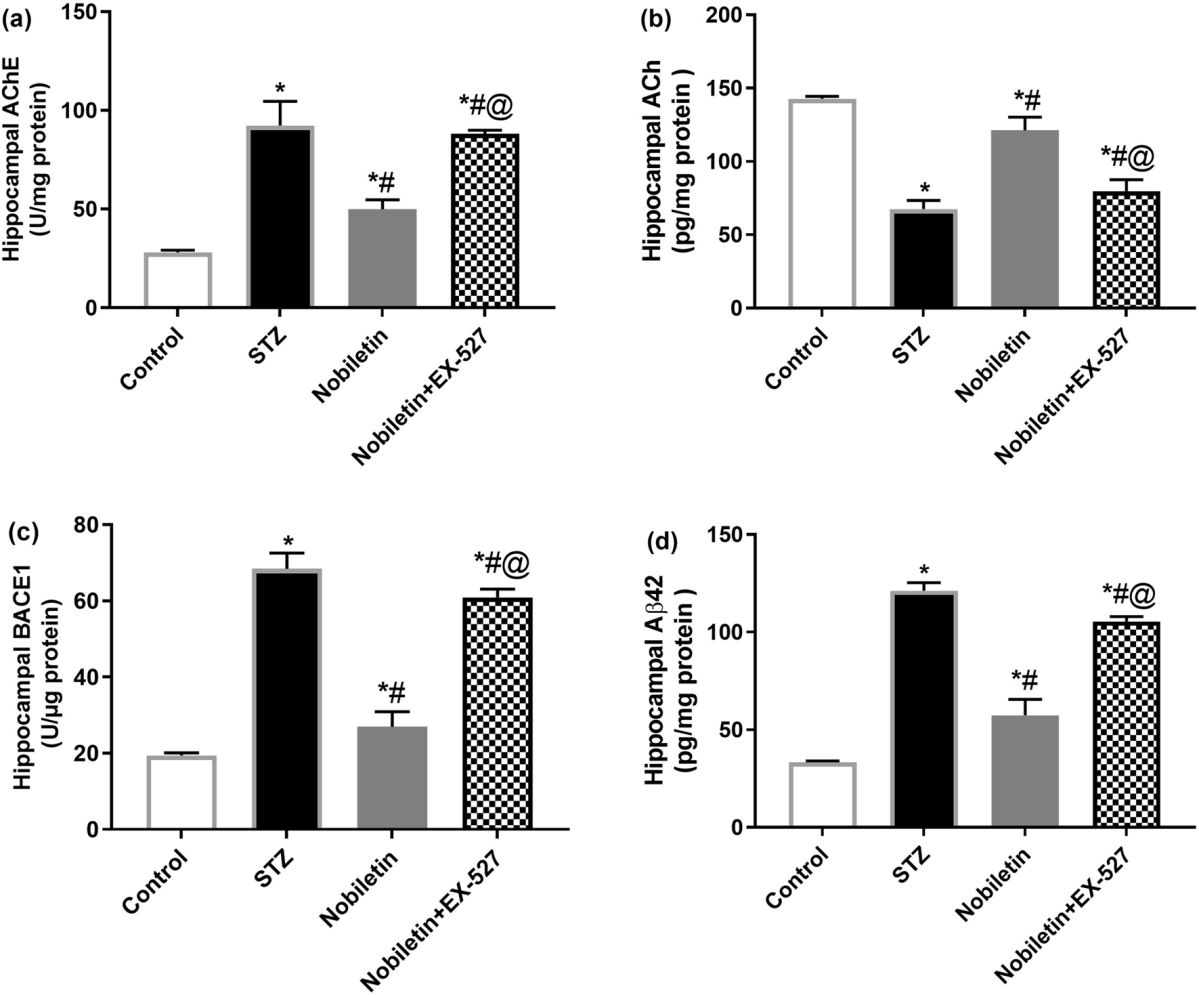
Administration of Nobiletin ameliorated STZ-induced changes in hippocampal **a** AChE enzyme activity, **b** ACh level, **c** BACE1 enzyme activity, and **d** Aβ42 level. The values are expressed as mean ± S.D. (*n* = 6) for each group. Using one-way ANOVA and Tukey’s post hoc test, values are statistically sig. at *p value < 0.05 vs. the control group, ^#^*p* value < 0.05 vs. the STZ group, and ^@^*p* value < 0.05 vs. the Nobiletin group

Moreover, the raise in BACE1 activity was lessened in Nobiletin-treated mice by 60.5% as compared to STZ-AD mice. STZ effect on Aβ42 was halted by Nobiletin administration which showed a 52% reduction, as compared to STZ mice. On the contrary, using EX-527 abolished the reductive effect of Nobiletin on AChE by 1.76-fold, while causing a 34.2% reduction in ACh levels as compared to Nobiletin, meanwhile EX-527 amplified BACE1 activity and Aβ42 levels by 2.25- and 1.83-fold, respectively (Fig. 2).

### Effect of Nobiletin on hippocampal SIRT1, p-Akt/t-Akt, FoxO3a, LC3B-I, LC3B-ll, and ATG7 in STZ-induced AD in mice

**Fig. 3:**
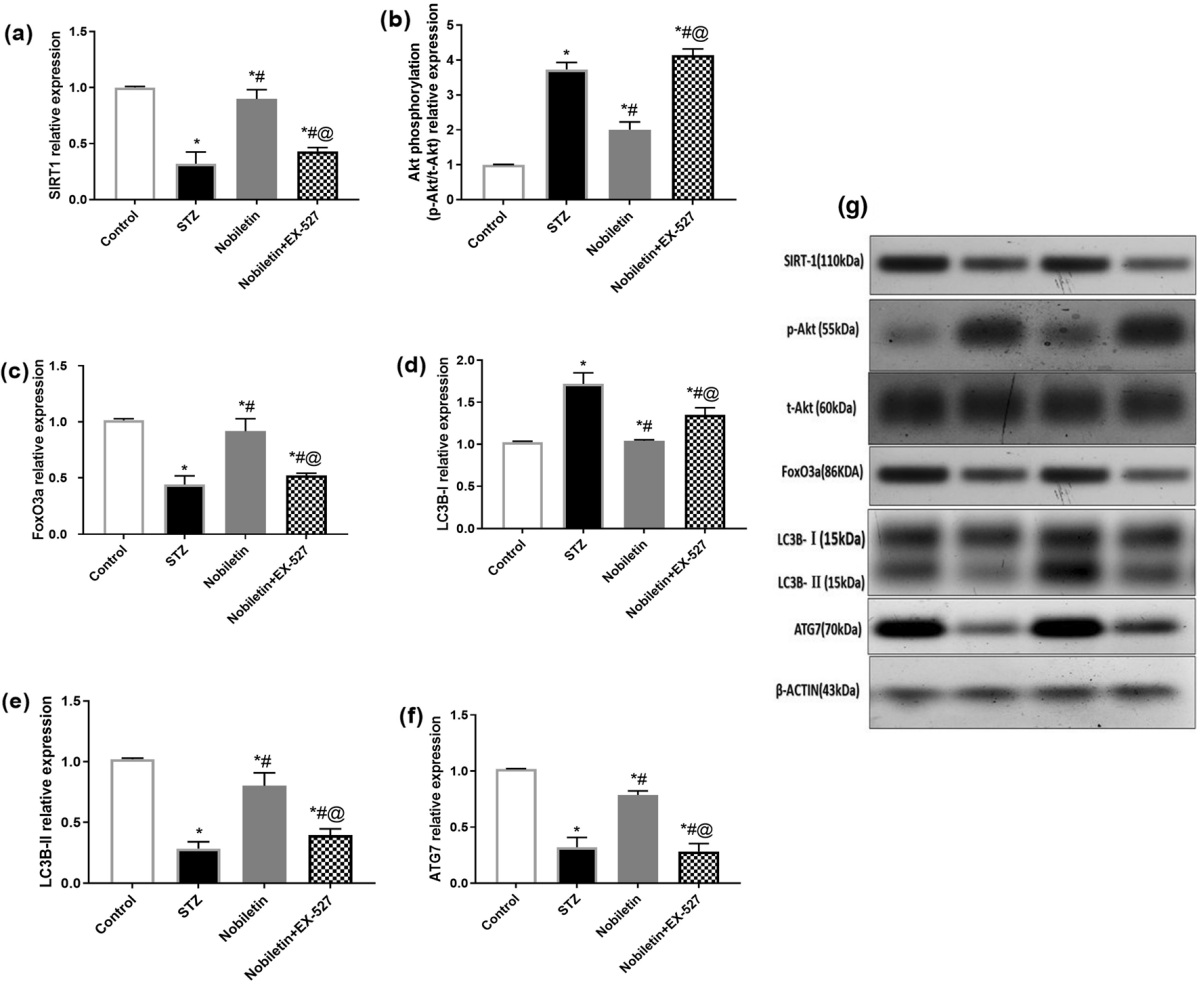
Quantification of **a** SIRT1, **b** p-Akt/t-Akt, **c** FoxO3a, **d** LC3B-I, **e** LC3B-II, and **f** ATG7 levels in the hippocampus relative to β-actin in different groups of mice using western blots. **g** Western blot bands of the respective parameters. The values are expressed as mean ± S.D. (*n* = 3) for each group. Using one-way ANOVA and Tukey’s post hoc test, values are statistically sig. at *p value < 0.05 vs. the control group, ^#^*p* value < 0.05 vs. the STZ group, and ^@^*p* value < 0.05 vs. the Nobiletin group

As shown in Fig. 3, compared to the control, STZ caused a dramatic decline in protein expression of hippocampal SIRT1, FoxO3a, LC3B-II, and ATG7 to nearly 68, 61, 71, and 69%, respectively, while causing approximately 70% and 280% increment in both hippocampal LC3B-I and Akt phosphorylation represented by p-Akt/t-Akt ratio, respectively, above their normal values. The autophagy activity was promoted significantly in Nobiletin-treated group as evidenced by the increase in SIRT1 (2.6-fold), FoxO3a (2.1-fold), LC3B-II (2.4-fold), and ATG7 (2.4-fold) as compared to STZ mice. In the meantime, these recoveries were dampened in the EX-527 pre-treated group by 50, 36, 42, and 66%, respectively, relative to Nobiletin group. Whereas Nobiletin showed a 47% reduction of p-Akt/t-Akt, and a 40% reduction in LC3B-I as compared to STZ group; however, EX-527 abolished this effect and showed enhancement of LC3B-I (1.25-fold) and p-Akt/t-Akt (2-fold).

### Effect of Nobiletin on hippocampal NF-κB and TNF-α levels in STZ-induced AD in mice

In STZ animals, NF-κB was accentuated by 2.3-fold from control base value and also its downstream effector, TNF-α (5-fold); however, administration of Nobiletin reduced NF-κB and TNF-α levels significantly by 48% and 59%, respectively, compared to the STZ group.

**Fig. 4:**
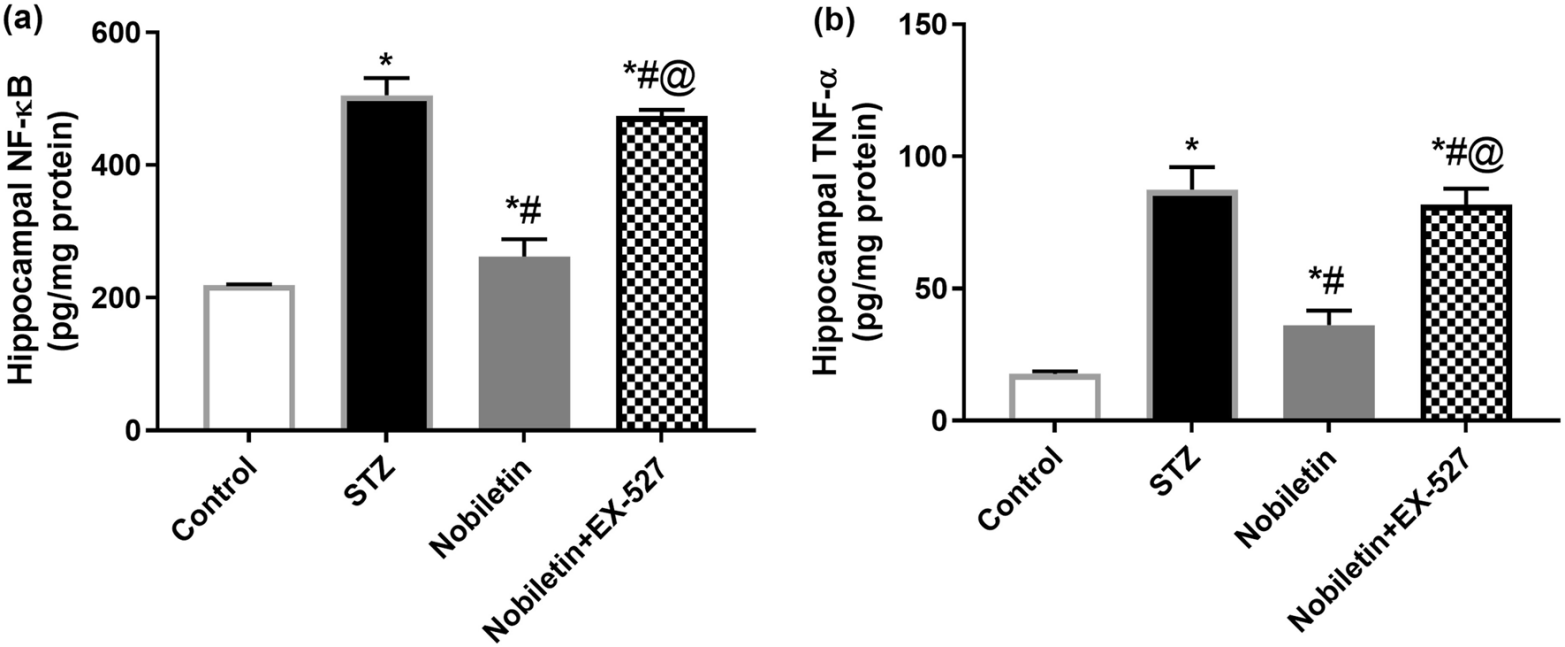
Administration of Nobiletin ameliorated STZ-induced changes in hippocampal, **a** NF-κB and **b** TNF-α levels. The values are expressed as mean ± S.D. (*n* = 6) for each group. Using one-way ANOVA and Tukey’s post hoc test, values are statistically sig. at *p value < 0.05 vs. the control group, ^#^*p* value < 0.05 vs. the STZ group, and ^@^*p* value < 0.05 vs. the Nobiletin group

Conversely, EX-527 administration diminished Nobiletin effect triggering an increment in NF-κB and TNF-α by 80.5% and 125.7%, respectively (Fig. 4).

### Effect of Nobiletin on hippocampal oxidative stress in STZ-induced AD in mice

**Fig. 5:**
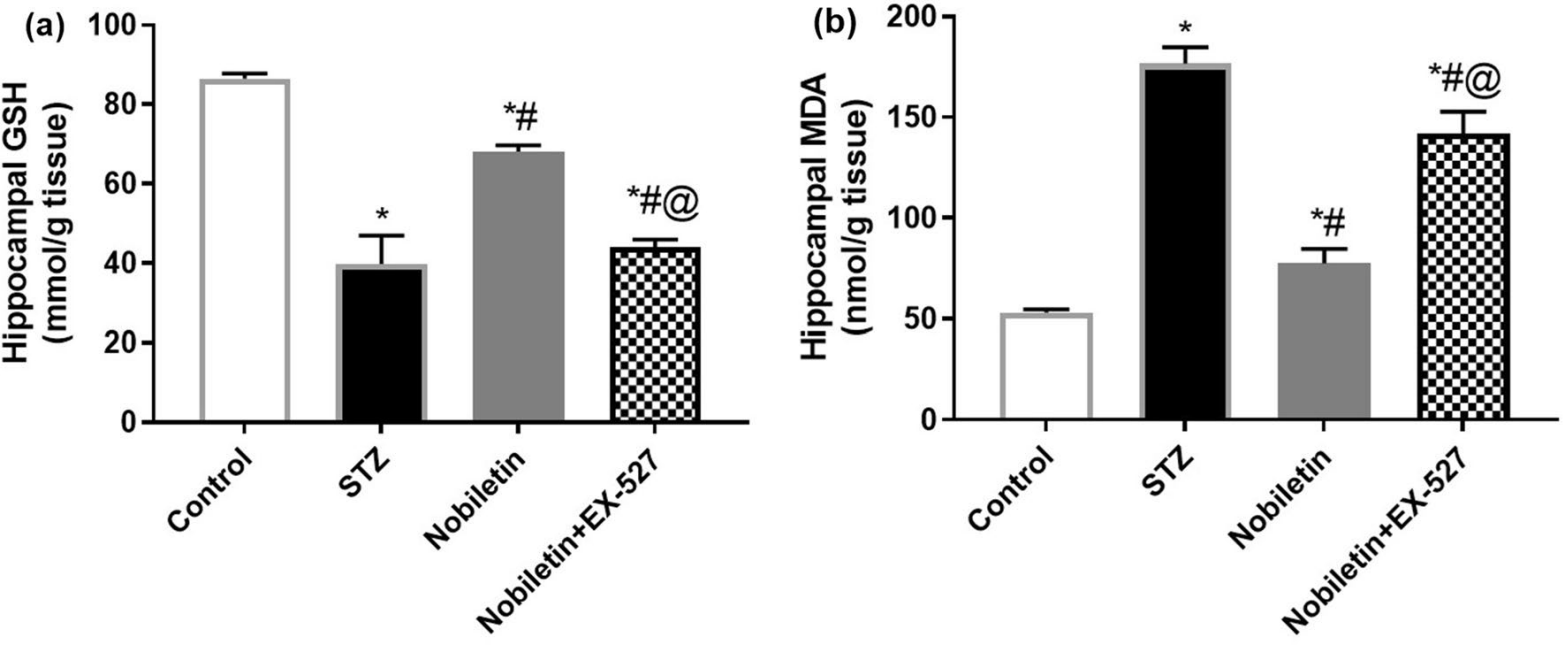
Administration of Nobiletin ameliorated STZ-induced AD changes in hippocampal **a** GSH and **b** MDA levels. The values are expressed as mean ± S.D. (*n* = 6) for each group. Using one-way ANOVA and Tukey’s Post hoc test, values are statistically sig. at *p value < 0.05 vs. the control group, ^#^*p* value < 0.05 vs. the STZ group, and ^@^*p* value < 0.05 vs. the Nobiletin group

ICV-STZ induced oxidative stress which was noticed by the significant upsurge in the level of MDA by 3.33-fold simultaneously with the diminution in reduced GSH level (54%) in hippocampus as contrasted to control values. Nobiletin successfully mitigated the glitch and restored the levels of MDA and reduced GSH by 56%, and 71% of STZ-AD induced mice. EX-527 pretreatment largely demolished Nobiletin-induced amendments and produced 1.8-fold enhancement in MDA level accompanied by 35% decrease in GSH level as compared to Nobiletin exposed ones as demonstrated in Fig. 5.

### Effect of Nobiletin on hippocampal histopathological alterations in STZ-induced AD in mice

**Fig. 6:**
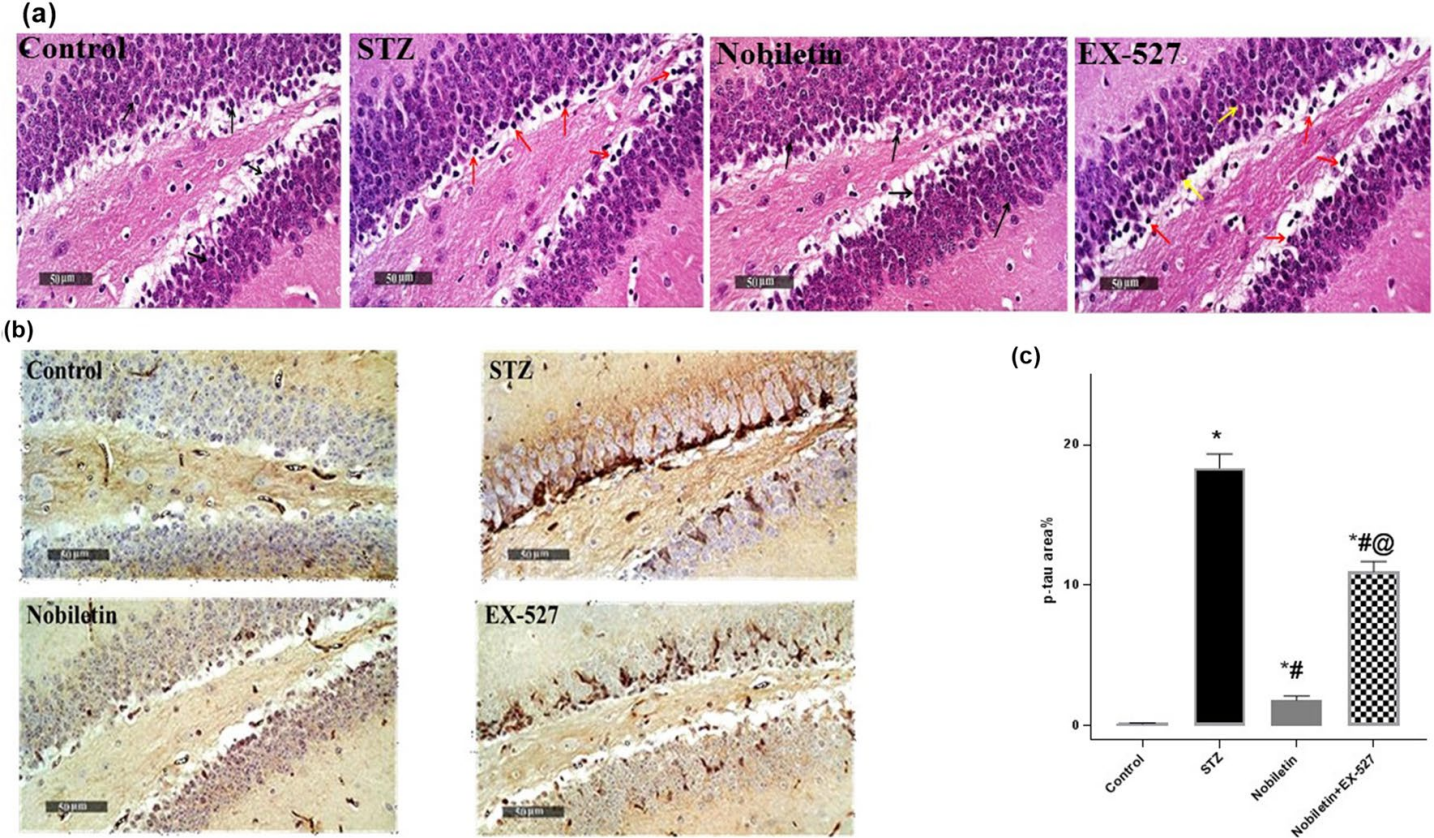
**a** Effect of Nobiletin on hippocampal histopathological alterations in dentate gyrus (DG) region in hippocampus in mice. Scale bars indicate 50 μm × 400. Normal histological characteristics of DG blades were seen in control samples. STZ model showed focal areas of degenerative changes of inner small granule cells with pyknotic nuclei (red arrows) as well as occasional scattered records of degenerated larger granule cells allover blades. Nobiletin group indicated well-organized morphological characteristics of DG region with many apparent intact granule cells without abnormal alterations (arrow). EX-527 group showed almost the same record as STZ model samples (red arrow). Moreover, scattered necrotic granule cells were observed (yellow arrow). **b** Immunohistochemically staining shows the changes in p-tau distribution patterns in the hippocampus. Scale bars indicate 50 μm × 400. **c** Quantitative data of p-tau aggregates. The values are expressed as mean ± S.D. (*n* = 3) for each group. Using one-way ANOVA and Tukey’s post hoc test, values are statistically sig. at *p value < 0.05 vs. the control group, ^#^*p* value < 0.05 vs. the STZ group, and ^@^*p* value < 0.05 vs. the Nobiletin group

As shown in Fig. 6a, normal morphological characteristics of hippocampus with evident intact well-organized neurons were exhibited in control samples. Contrariwise, photomicrographs from STZ mice demonstrated degeneration in focal areas of inner small granule cells with pyknotic nuclei as well as occasional scattered degenerated larger granule cells allover blades. Nobiletin group, however, showed almost well-organized hippocampal region and morphological features of DG region with many apparent intact granule cells without abnormal alterations. Pre-administration of EX-527 negated Nobiletin effect and, interestingly, mice from this group showed almost the same records as STZ model samples and scattered necrotic granule cells were observed.

### Effect of Nobiletin on hippocampal p-tau levels in STZ-induced AD in mice

As shown in Fig. 6b–c, normal mice did not show any p-tau staining. On the contrary, numerous aggregates were observed in STZ-induced AD mice, showing 141-fold increase in hippocampal p-tau immunoreactivity when compared to the normal which is a fundamental marker for AD. Nobiletin hindered p-tau aggregations by 90% as compared to STZ-induced AD mice, elaborating that Nobiletin can improve AD in vivo. Marked increase, of about 6.1-fold in p-tau appears in group pre-treated with EX-527 when compared to Nobiletin group.

## Discussion

The current study emphasizes, for the first time, the incentive impacts of Nobiletin as a neuroprotective candidate against AD in an STZ animal model. The following conclusions can be drawn (i) enhanced learning, memory, as well as cognitive improvement; (ii) autophagy induction through SIRT1/FoxO3a as well as Akt/FoxO3a signaling pathways; (iii) impeding cholinergic neurodegeneration observed through the inhibition of AChE activity; (iv) attenuating neuronal cell injury via marked reduction in Aβ42 and p-tau; (v) reduced neuroinflammation indicated by mitigating NF-κB and its downstream effector, TNF-α; (vi) reduced oxidative stress manifested by upsurge in GSH and decreased MDA levels.

The long-term gradual deterioration in learning, memory, and cognitive behavior in a mouse model stimulated by ICV-STZ mimics the phases of Alzheimer's disease (Hindam et al. 2020; Nazem et al. 2015). Currently, ICV-STZ administration showed a decline in spatial learning, cognition, long-term memory and working memory as demonstrated by changes in the behavioral tests. These results are consistent with others (Halawany et al. 2017; Rasheed et al. 2018; Souza et al. 2017). Moreover, impairment in working memory observed in ICV-STZ mice was confirmed by the increase in the alternation percentage using Y-maze assay, a result that is in line with Ghoneum (2021). The increased latency time and the short time spent in the target quadrant of the MWM test reported in this research were also similar to those described by other authors (Li et al. 2016; Thomé et al. 2018). On the contrary, Nobiletin enhanced both recognition and discrimination indices in ORT. A prior study showed the influence of Nobiletin on behavior in the triple-transgenic mouse model of AD mice, which agrees with our findings (Nakajima et al. 2015). Likewise, Nobiletin was reported to enhance memory in olfactory bulbectomy mice (Nakajima et al. 2007).

Obvious neurological impairment in the hippocampal region is an illustrious characteristic of AD (Tiwari et al. 2009). The existing histopathological findings revealed that STZ triggered immense neuronal deterioration and gliosis in the DG region of the hippocampus in addition to discernible neuronal damage as evinced by H&E staining. Along parallel lines, histopathological results propose that STZ promotes structural as well as functional destruction of hippocampal regions in rodents (Salkovic-Petrisic et al. 2006; Ponce-Lopez et al. 2017; Liu et al. 2013a). Nobiletin, on the other hand, safeguarded the neurons from inflammation-related damage induced by STZ and enhanced neuronal cell survivability. Consistent with these observations, a study previously reported that Nobiletin protected mice from neuronal death, resulting in the survival of 50% of the neurons in the hippocampus (Yamamoto et al. 2009).

In the current study, STZ mastered the occurrence of AD through three major mechanisms, beginning with its effect on AChE which led to cholinergic deficiency and cognitive impairment. Moreover, the effect on BACE-1 enzyme ensuing an increase in oxidative stress, proinflammatory cytokines, and hippocampal histopathological changes in mice, ending with inhibition of autophagy because of STZ effect on SIRT1.

Starting with the first mechanism, ICV-STZ mice showed an increment in the hippocampal AChE activity which rapidly hydrolyzed ACh leading to loss of cholinergic neurons. AChE is currently thought to be a necessary molecular chaperon of Aβ aggregation. In the brain of people with AD, AChE is localized in amyloid deposits and the mature senile plaques (Morán and Mufson 1993). Furthermore, researches have proved that AChE is capable of aggravating Aβ toxicity through creating a settled AChE–Aβ complex (Luo et al. 2011). Accumulation of Aβ implies suppression and internalization of nicotinic acetylcholine receptor (α7 nAChR) which induces a negative feedback mechanism (Fabiani 2019). This Aβ-α7 complex also affects tau hyperphosphorylation, which results in synaptic dysregulation and memory problems (Wang and Li 2003). By lowering AChE activity in the hippocampus, Nobiletin had a positive impact on ACh level, thus retaining memory and cognition. Previous studies have reported findings that are consistent with ours that proved the anti-cholinesterase effect of Nobiletin (Nakajima and Ohizumi 2014; Nakajima et al. 2007).

The second pathway of ICV-STZ-induced cognitive in-capabilities in mice is the augmentation of BACE-1 enzyme level that induces amyloid precursor protein (APP) sequential proteolytic cleavage resulting in the excessive production of Aβ in neurons. The study conducted by Liu et al*.* (2013b) showed elevation in Aβ1-40/Aβ1-42, APP, and BACE1 levels upon injection of STZ, results that are in harmony with ours. Moreover, the marked dispersion of Aβ1-42 peptide in the hippocampus attributed to the significant escalation in oxidative stress and concomitantly, neuroinflammation (Zameer et al. 2019). Indubitably, the production of reactive oxygen species plays a crucial role in the pathogenesis and progression of AD (Correa et al. 2012; Grammas 2011). This was evidenced, in the current study, by a significant upsurge in MDA levels, an end product of lipid peroxidation, as well as depletion in GSH content. Our results correspond with the previous experimental studies that signify the origination of free radicals as a foremost determinant of STZ neurotoxicity (Tota 2011; Saxena et al. 2007). Furthermore, the deleterious influence of oxidative stress extends to hyperphosphorylation of tau protein, as reported in our study, via glycogen synthase kinase-3β (GSK-3β) phosphorylation which is correlated with the development of memory and learning impairment in STZ mice (Saxena et al. 2008). On the contrary, Nobiletin administration halted the activity of BACE-1 enzyme which contributes to the attenuation of Aβ peptide accumulation in the hippocampus displayed by a significant lower level of Aβ42 (Youn et al. 2017). Indeed, Nobiletin’s anti-oxidant features (Nakajima et al. 2007; Nakajima and Aoyama 2013) were noted by the significant alleviation of oxidative stress as revealed by decreased MDA levels accompanied by GSH restoration.

In addition, oxidative stress acts as a paramount inducer of proinflammatory mediators in neurodegenerative disorders through several mechanisms. One of which is induction of NF-κB directly through activation of inhibitor of nuclear factor-kappa B kinase subunit beta (IKK-β) which phosphorylates IκB protein leading to its degradation and subsequent liberation of NF-κB (Evans et al. 2003). Moreover, it can indirectly activate NF-κB through PI3K stimulation, which upon phosphorylation, PI3K activates Akt (Hemmings 2012). The latter can phosphorylate and stimulate IKK, hence activating NF-κB (Bai et al. 2009). Once activated, NF-κB translocates into the nucleus and increases the transcription of inflammatory mediators such as TNF-α (Williams and Ozment-Skelton 2006). In the present work, STZ injection led to a substantial elevation in NF-κB and TNF-α levels in the hippocampus. However, Nobiletin administration hindered the inflammatory process through reducing the levels of NF-κB and TNF-α confirming the anti-inflammatory potential of Nobiletin. These results were in accordance with other studies that showed the ability of Nobiletin to repress the activation of NF-κB which could be related, in part, to inhibiting PI3K/Akt pathway and its downstream genes (Cui et al. 2010; Qi et al. 2019). The third mechanism of STZ-induced pathology is the impediment of SIRT1 activity. SIRT1 activation may be a good potential strategy to counteract amyloid deposition and neurodegeneration in AD (Pasinetti et al. 2011). SIRT1 deficiency was much more prominent in AD patients (Kumar et al. 2013). The beneficial effect of SIRT1 could be related to being a modulator of autophagy both directly by promoting the deacetylation of autophagy genes ATG5, ATG7, and ATG8 and indirectly by regulating FoxO3a transcription factor which, in turn, controls the expression of several pro-autophagic proteins (Lewandowska et al. 2016). The nuclear translocation of FoxO3a promotes the expression of autophagy-correlated proteins such as LC3B, which plays a role in autophagosome formation (Hariharan et al. 2010). Conjugation of LC3-I to phosphatidylethanolamine to form LC3B-phosphatidylethanolamine conjugate (LC3B-II) escorts autophagosomes assembly and activity. The relation of LC3B-II to LC3B-I levels dependably signifies the level of autophagic activity (Naik et al. 2018). In this study, STZ injection caused profound detraction of SIRT1, FoxO3a expression, along with ATG7 and LC3B-II proving the detrimental effect of inhibiting autophagy in AD. The reduction in SIRT1 activity could be linked to the imbalance in NAD/NADH ratio in the hippocampus of the rodents which was reported as an important regulator for sirtuin activity in previous studies (Du et al. 2014; Bordone 2005). Surprisingly, the impact of SIRT1 on the initial stages of tauopathy is inevitable. SIRT1 deacetylates tau leading to its degradation (Min et al. 2010) which was evidenced in our study as increased expression of p-tau as a result to decreased SIRT1 activity. Meanwhile, treatment with Nobiletin upregulated SIRT1 expression and increased the nuclear translocation of FoxO3a leading to elevation of ATG7 and LC3B-II, the autophagy-related genes. Moreover, Nobiletin, as an activator of SIRT1 signaling pathways, deacetylated p-tau, hence preventing tauopathy. Remarkably, our results are in concordance with former studies revealing tau pathology is exceedingly reflective of cognitive decline in AD indicating that Nobiletin may be an efficient therapy targeting phosphorylated tau and preceding to cognitive enhancement (Berg et al. 1998; Arriagada et al. 1992). Previous studies have reported a similar response of animal models to Nobiletin as a therapeutic potential for AD and other neurodegenerative disorders, which supports these findings (Nakajima and Ohizumi 2014; Qi et al. 2019; Onozuka et al. 2008).

Interestingly, the link between Akt and autophagy cannot be concealed where earlier research has found that elevated levels of p-Akt (Ser473) results in autophagy inhibition as FoxO3a can be directly phosphorylated by Akt and becomes sequestered in the cytoplasm (Fasano et al. 2019). This suppresses FoxO3a transcriptional action by causing a significant decrease in nuclear FoxO3a level and presumably diminished autophagy regulatory gene expression (Im and Hergert 2015). Moreover, p-Akt (Ser473) led to phosphorylation and activation of GSK3β, the major tau kinase (Griffin et al. 2005). STZ injection in the current experiment showed augmented phosphorylation of Akt, an effect that was reported in past studies indicating that brain autophagy was dampened in STZ-treated mice (Bordone 2005; Agrawal et al. 2011) On the other hand, Nobiletin depressed the level of p-Akt (Ser473), as previously mentioned (Chen et al. 2014; Lee et al. 2011), and thus stimulating autophagy and halting the progression of AD.

This triad emphasizes the possible pathway of STZ to induce AD and the relation between STZ, AD, and autophagy. As well, the influence of Nobiletin as a promising therapy to tolerably increase autophagy which may indeed be beneficial for AD treatment.

Intriguingly, to inspect whether SIRT1 activity is demanded for Nobiletin-mediated amelioration of AD, a specific SIRT1 inhibitor (EX-527) was used. SIRT1 is inhibited by EX-527 through blocking SIRT1’s NAD^+^ binding site resulting in selectively inhibition of SIRT1 activity (Vachharajani et al. 2014; Gertz et al. 2013). Administration of EX-527 led to the loss of Nobiletin-mediated improvement in memory and learning. In addition, EX-527 reversed the neuroprotection of Nobiletin on STZ-induced AD as evidenced by behavioral, biochemical, histopathological, and immunohistochemical results. These data have suggested that the effect of Nobiletin on STZ-induced AD was mediated through a SIRT1-dependent mechanism.

Taken all together, disruption of autophagy may play an important role in several neurodegenerative disorders, such as AD. Hence, autophagy augmenting therapies could emerge in treating neurodegenerative diseases. Consequently, Nobiletin-induced autophagy may be an ultimate strategy for reducing amyloid deposition, and neurotoxicity in the AD brain, as evidenced by our findings. In addition, Nobiletin conquered AD through several mechanisms including anti-oxidant, anti-inflammatory (Nakajima and Ohizumi 2019), enhancing autophagy through SIRT1/FoxO3a pathway, cognitive and behavioral preservation, halting cholinergic neurodegeneration as well as Aβ pathology, and tau hyperphosphorylation. These findings suggest that this natural substance could be developed into a novel anti-Alzheimer drug.

## Conclusion

Nobiletin ameliorates neurodegeneration in a mouse model of STZ-induced AD via stimulating autophagy through activating SIRT1/FoxO3a pathway, blocking the phosphorylation of Akt and its inhibiting downstream targets to control the inflammatory response and oxidative stress, and to control the cholinergic system. Nobiletin restored the incapacitated autophagy flux demonstrated by the diminished LC3B-II and ATG abundance. Therefore, Nobiletin could offer a promising therapeutic strategy for AD.
